# Comparison Study of the Safety Profile of Olaparib Versus Niraparib: Analysis of Real-World Data from EudraVigilance

**DOI:** 10.3390/ph18040528

**Published:** 2025-04-04

**Authors:** Desirèe Speranza, Fausto Omero, Vincenzo Cianci, Mariapia Marafioti, Carla Infurna, Patrizia Carroccio, Edoardo Spina, Maria Antonietta Barbieri, Emanuela Esposito, Nicola Silvestris, Mariacarmela Santarpia

**Affiliations:** 1 Department of Chemical, Biological, Pharmaceutical and Environmental Sciences, University of Messina, 98166 Messina, Italy; desiree.speranza@gmail.com (D.S.); emanuela.esposito@unime.it (E.E.); 2 Department of Human Pathology “G. Barresi”, School of Specialization in Medical Oncology, University of Messina, 98125 Messina, Italymarafiotimariapia@gmail.com (M.M.); karla.infurna@hotmail.it (C.I.);; 3 Department of Biomedical and Dental Sciences and Morphofunctional Imaging, Section of Legal Medicine, University of Messina, 98125 Messina, Italy; enzocianci.1997@gmail.com; 4 Department of Clinical and Experimental Medicine, University of Messina, 98122 Messina, Italymariaantonietta.barbieri@unime.it (M.A.B.); 5 Medical Oncology Unit, IRCCS Istituto Tumori “Giovanni Paolo II”, 70124 Bari, Italy; 6 Medical Oncology Unit, Department of Human Pathology “G. Barresi”, University of Messina, 98122 Messina, Italy

**Keywords:** olaparib, niraparib, EudraVigilance, pharmacovigilance, PARP inhibitors, real-world data, safety profile, ovarian cancer

## Abstract

**Background:** Olaparib and niraparib are poly (ADP-ribose) polymerase inhibitors (PARPi) used primarily for the treatment of ovarian cancer. While both drugs have demonstrated efficacy in clinical trials, their safety profiles, particularly in real-world clinical settings, remain to be fully elucidated. **Objectives:** This study aimed to (i) characterize the adverse drug reactions (ADRs) associated with olaparib and niraparib as reported in the EudraVigilance database, (ii) compare the frequency of the ADRs occurring during treatment with the two drugs, and (iii) compare post-marketing safety data with those from clinical trials. **Methods:** A retrospective analysis was performed using data from the EudraVigilance database (2017–2024), focusing on individual case safety reports (ICSRs) related to olaparib and niraparib. Descriptive statistics and disproportionality analysis were performed to compare the frequency and severity of reported ADRs. **Results:** Both olaparib and niraparib had common ADRs including nausea, vomiting, anemia, thrombocytopenia, and fatigue. However, olaparib was associated with a higher risk of myelodysplastic syndrome (MDS), acute myeloid leukemia (AML), and interstitial lung disease, while niraparib had a higher incidence of gastrointestinal events and thrombocytopenia. Our analysis demonstrates that some specific ADRs, including peripheral neuropathy with niraparib, were reported at higher frequencies compared to clinical trials. The incidence of serious ADRs, including hospitalizations and life-threatening events, was higher with niraparib than with olaparib. **Conclusions:** This study highlights significant differences in the safety profiles of olaparib and niraparib, with implications for clinical decision-making. Continuous monitoring and personalized management of ADRs are essential to optimize patient outcomes.

## 1. Introduction

Ovarian cancer (OC) represents 3.4% of all cancer cases and is the eighth leading cause of cancer-related death among the female population worldwide. Although it can arise from different cell types, epithelial OC is the most common form [1]. Molecular characterization of ovarian carcinomas has allowed the development of several targeted agents, such as PARPi, that have markedly changed the therapeutic landscape of these tumors in recent years.

It is well established that mutations in the *BRCA1* and *BRCA2* genes are strongly associated with the occurrence of ovarian cancer, both in sporadic and familial forms. These genes play a pivotal role in the repair of damaged DNA through the homologous recombination (HR) pathway and non-homologous end joining (NHEJ) pathways [2]. When mutated, cells become more susceptible to the accumulation of genetic damage, thus increasing the risk of developing cancer.

Over the past years, different PARPi, including olaparib (OLA) and niraparib (NIRA), have been developed and approved for clinical use in different settings for this disease.

Given the increasing utilization of PARPi for OC, there is an urgent need to acquire more data on their efficacy and toxicity, outside clinical trials, to improve patient selection and the management of adverse events (AEs). The objective of this study is to describe the adverse reactions of OLA and NIRA that have been reported and collected in EudraVigilance (EV). To this end, a comparison of the safety profiles of NIRA and OLA is presented. Finally, the real-world data are compared with the safety profiles of the drugs reported in the literature.

### 1.1. Mechanisms of PARP Inhibition in DNA Repair

PARP enzymes, particularly PARP1 and PARP2, are pivotal in the repair of SSBs via the base excision repair (BER) pathway. Under normal conditions, PARP binds to damaged DNA and catalyzes the addition of poly-ADP-ribose chains (PARylation), which facilitates the recruitment of DNA repair factors, such as XRCC1 and DNA ligase III, to promote the repair of single-strand breaks (SSBs) (2) specifically. PARPi, including OLA and NIRA, bind to the NAD+ binding site of PARP1/2, preventing their enzymatic activity and subsequent PARylation. This blockage leads to the accumulation of unrepaired SSBs, which are converted into double-strand breaks (DSBs) during DNA replication. DSBs are far more detrimental to cell survival and require repair through HR or NHEJ pathways [2].

In the context of DSBs, the MRN complex (comprising Mre11, Rad50, and Nbs1) is crucial for the detection, signaling, and repair of these lesions. Upon DSB formation, the MRN complex is recruited to the site of damage, where it performs several critical functions. These include the processing of the break, resection of the DNA ends, and activation of ATM kinase, which is essential for signaling DNA damage and initiating repair pathways [2]. In particular, the MRN complex plays a role in the early steps of HR by facilitating the generation of single-stranded DNA (ssDNA) overhangs, which are necessary for HR-mediated repair. The MRN complex also helps in tethering the broken DNA ends together, preventing chromosomal fragmentation. Importantly, the MRN complex is involved in the “bridging” of broken DNA ends in the NHEJ repair pathway, which may operate in parallel with HR, especially in HR-deficient cells [3].

In cells with intact homologous recombination repair (HRR), such as those without *BRCA* mutations, DSBs can be repaired efficiently, and the cells remain viable. However, in cancer cells with HRR deficiencies—most notably those with *BRCA1* or *BRCA2* mutations—the repair of DSBs is compromised. This results in the accumulation of irreparable DNA damage, which ultimately leads to cell death [2,3].

**Figure 1:**
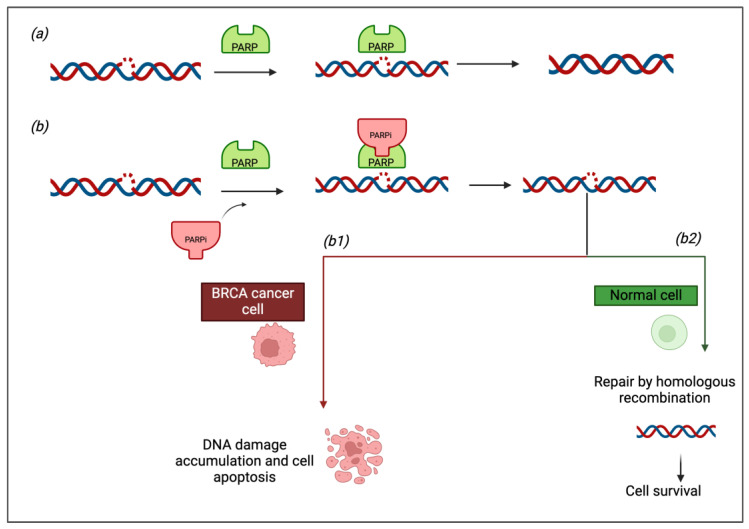
The principle of synthetic lethality: the focus is on exploiting the weakness of *BRCA*-deficient cancer cells. In a normal cell (**a**), when DNA damage occurs, the cell activates DNA repair mechanisms, such as homologous recombination, to fix the damage. However, in *BRCA*-deficient cancer cells (**b**), this crucial repair pathway is impaired. When a PARP inhibitor is introduced, it blocks the activity of PARP enzymes, which are essential for repairing single-strand DNA breaks. In normal cells, this inhibition is not a significant problem, as they can still rely on homologous recombination for repair (**b2**) (green arrow). However, in *BRCA*-deficient cancer cells, the inability to repair DNA damage through both pathways leads to the accumulation of DNA damage and, ultimately, cell death (**b1**) (red arrow).

The concept of “synthetic lethality” underpins the therapeutic strategy of PARPi, particularly in OC with homologous recombination deficiency (HRD) (i.e., *BRCA1/2* mutated) (Figure 1). In cells with defective HR, the inhibition of PARP exacerbates DNA damage by preventing SSB repair and promoting the formation of DSBs, a type of lesion that these cells are unable to repair due to the lack of functional BRCA proteins. This causes selective toxicity in cancer cells with HRR deficiencies, while sparing normal cells with intact HR [3].

### 1.2. Summary of Clinical Efficacy of PARP Inhibitors in Ovarian Cancer

First, NIRA and OLA FDA approval for use against OC was obtained in second-line or later maintenance settings, based on phase III NOVA and SOLO2 trial results. In the NOVA study, NIRA was evaluated as maintenance treatment in recurrent, platinum-sensitive OC. Progression-free survival (PFS) was longer compared to the placebo, regardless of the presence or absence of germline *BRCA* (gBRCA) mutations or HRD status, with moderate toxicity (thrombocytopenia 33.8%, anemia 25.3%, and neutropenia 19.6%). The SOLO2 trial studied the efficacy of OLA as maintenance therapy for relapsed, platinum-sensitive, *BRCA*-mutated OC; mPFS was significantly longer in the experimental arm (19.1 vs. 5.5 months, *p* < 0.0001) than with the placebo; bone marrow toxicity was common, with grade 3 anemia in 19% of patients who received OLA [4,5].

Olaparib was the first PARPi to be approved as first-line maintenance monotherapy, based on the results of the phase III SOLO1 trial, in which 391 randomized patients with *BRCA*-mutated, advanced (International Federation of Gynecology and Obstetrics, FIGO, stage III or IV), high-grade serous or endometrioid ovarian, fallopian tube, or primary peritoneal cancers, who had a clinical response (complete or partial) to platinum-based chemotherapy received OLA or placebo for up to 2 years or until progression (PD). The trial showed clinical benefit both in mPFS, the primary endpoint, (56.0 vs. 13.8 months at 5-year follow-up, HR 0.33, 95% CI 0.25–0.43), and in OS (NR vs. 75.2 months at seven-year follow-up, HR 0.55, 95% CI 0.40–0.76) [6,7,8].

The phase III PRIMA trial enrolled 733 patients with advanced, high-grade serous or endometrioid ovarian, fallopian tube, or primary peritoneal cancers, regardless of *BRCA* status and who responded to first-line platinum-based chemotherapy, to receive maintenance NIRA for 3 years or until PD. Updated mPFS at 3.5-year follow-up was 13.8 vs. 8.2 months (HR 0.66, 95% CI 0.56–0.79, *p* < 0.0001) in the overall population, with greater benefit in the HRD-positive subgroup (PFS 24.5 vs. 11.2 months) [9,10]. Based on these results, NIRA obtained FDA approval as a maintenance therapy in the first-line setting, irrespective of biomarker status.

The randomized PAOLA-1 and OVARIO evaluated the efficacy of PARPi in association with bevacizumab (BEVA) as a first-line maintenance therapy for OC. Adding BEVA to OLA in the maintenance setting (PAOLA-1) resulted in longer PFS compared to with placebo and BEVA (median 22.1 vs. 16.6 months; *p* < 0.001). A median PFS2 of 36.5 months vs. 32.6 months in the control arm (*p* = 0.0125) was observed. Overall, better survival outcomes were observed in patients with *BRCA*-mutated or HRD-positive tumors, leading to FDA approval of the combination in these patients. In the phase II OVARIO study, NIRA-BEVA led to encouraging PFS results (18-month PFS rate of 62% in overall population, 76% in HR deficient group, and 47% in HR proficient group) [11,12].

According to the 2023 ESMO guidelines for EOC, PARPi are recommended as maintenance therapy with the following indications: OLA as a monotherapy for *BRCA 1/2*-mutated EOC, and OLA in combination with BEVA for HRD-positive EOC [13]. Additionally, in September 2020, the EMA approved NIRA for use, regardless of biomarker status (*BRCA* or HRD) [14]. For platinum-sensitive recurrent EOC, PARPi can be used as maintenance therapy regardless of *BRCA* or HRD mutation status. The 2024 NCCN guidelines indicate PARPi for EOC patients with high-grade serous, grade 2/3 endometrioid, and *BRCA 1/2*-mutated clear cell carcinoma, or carcinosarcoma [15]. The decision to prescribe PARPi should be individualized, taking into account factors like the use of BEVA in the primary therapy, *BRCA 1/2* status, and radiological response to initial chemotherapy. Niraparib may be prescribed for patients who have not received BEVA as first-line therapy, have *BRCA* 1/2 wild-type or unknown status, and have achieved a complete or partial response. Treatment can continue until PD, the onset of unacceptable toxicity, or for up to three years. Olaparib is recommended for patients with a germline or somatic *BRCA 1/2* mutation and a complete or partial response, with treatment continuing until PD, unacceptable toxicity, or for up to two years. The FDA approved first-line maintenance OLA on December 19, 2018, and NIRA on April 29, 2020 [16,17]. Patients who received BEVA as part of their primary therapy may continue maintenance treatment with either BEVA plus OLA or BEVA plus NIRA, provided they are *BRCA 1/2* wild-type or have an unknown status, are HR-deficient, and have had a complete or partial radiological response. This can continue until PD or unacceptable toxicity, with a maximum of three years for NIRA or two years for OLA.

## 2. Results

### 2.1. Literature Review

In order to evaluate the safety of NIRA, three randomized controlled trials (RCTs) and one phase II trial were considered, involving a total of 2014 patients. According to the NORA study, all patients experienced treatment-related adverse events (TRAEs), with the most prevalent being a decrease in white blood cell count (59.3%), neutrophil count (58.8%), and platelet count (54.8%), anemia (53.1%), nausea (53.1%), vomiting (32.2%), and constipation (29.9%) [18]. The incidence of TRAEs of grade 3 or higher was 50.8% in patients treated with NIRA, compared to 19.3% in those treated with the placebo. All AEs were managed through dose reduction or treatment interruption [18,19]. In the NOVA study, the AEs observed with greater frequency in the NIRA arm included thrombocytopenia (28.3%), anemia (24.2%), neutropenia (11.2%), hypertension (8.2%), and fatigue (5.7%) [4]. Furthermore, the PRIMA study demonstrated a high prevalence of hematological AEs, particularly during the initial month of therapy. In this study, the incidence of grade ≥3 AEs in the NIRA arm were as follows: thrombocytopenia (39.7%), anemia (31.6%), and neutropenia (21.3%) [20,21]. Moreover, in the same RCT, the incidence of MDS or AML was comparable between the NIRA and placebo arms, with a prevalence of 1.2% in each group [21]. In the QUADRA trial, documented TRAEs included nausea (58%), vomiting (32%), constipation (17%), anemia (44%), thrombocytopenia (33%), fatigue (41%), and a decrease in platelet count (21%) [22]. The incidence of TRAEs of grade 3 or higher was 24% for anemia and 21% for thrombocytopenia. Severe TRAEs were observed in 7% of patients, with the most common being small intestinal obstruction, thrombocytopenia, and vomiting. The management of AEs involved a reduction in dose (62%), treatment interruption (47%), and discontinuation (21%). The most severe TRAE was a fatal gastric hemorrhage [4,6,9] (Table 1).

The safety of OLA was evaluated based on the findings of three RCTs and one phase II trial, with a total of 1080 patients enrolled. The AEs documented in the SOLO 1 and SOLO 2 studies were as follows: nausea (77.7% and 75.9%), fatigue (64.2% and 65.6%), anemia (40% and 43.6%), vomiting (40% and 37.4%), and neutropenia (23.1% and 19%) [5,7,8]. Additionally, diarrhea was observed in 34.6% of patients in SOLO 1 and 32.8% in SOLO 2. The incidence of AEs graded as 3 or higher was 21.2% in SOLO 1 and 36.9% in SOLO 2. Adverse events were managed by reducing or interrupting therapy, with the majority of treatment interruptions occurring in the OLA arm, compared to the placebo arm [5,7,8].

Both studies documented an elevated risk of MDS or AML in the OLA arm relative to the placebo arm. The incidence of other cancers (lung, tongue, breast, lips, thyroid, pancreas, and gallbladder) was observed in 4.5% of patients treated with OLA and 5.6% of those treated with placebo. In the SOLO 3 study, the most commonly reported AEs in patients receiving OLA were nausea, fatigue/asthenia, anemia, vomiting, and diarrhea [23]. A total of 2.2% of patients in the OLA arm developed MDS or AML. Fatal AEs observed in the OLA cohort included MDS, cardiopulmonary decompensation, AML, subarachnoid hemorrhage, and sepsis. The primary reasons for therapy cessation were emesis, anemia, and thrombocytopenia [23].

In the LIGHT trial, 98.5% of patients experienced AEs, with 43.5% of these classified as grade 3 or higher. The most prevalent AEs were nausea (66.4%), fatigue/asthenia (62%), vomiting (32.8%), and anemia (28.8%) (Table 2). Resolution of AEs was verified within a 12-month period. Treatment interruption was documented in 33.2% of patients, dose reduction in 24.4%, and treatment discontinuation in 4.4% [24].

### 2.2. Real-World Data Analysis

A total of 11,259 reports with NIRA and OLA as suspected drugs was collected from January 2017 to April 2024, with the majority related to NIRA (*n* = 6385).

**Figure 2:**
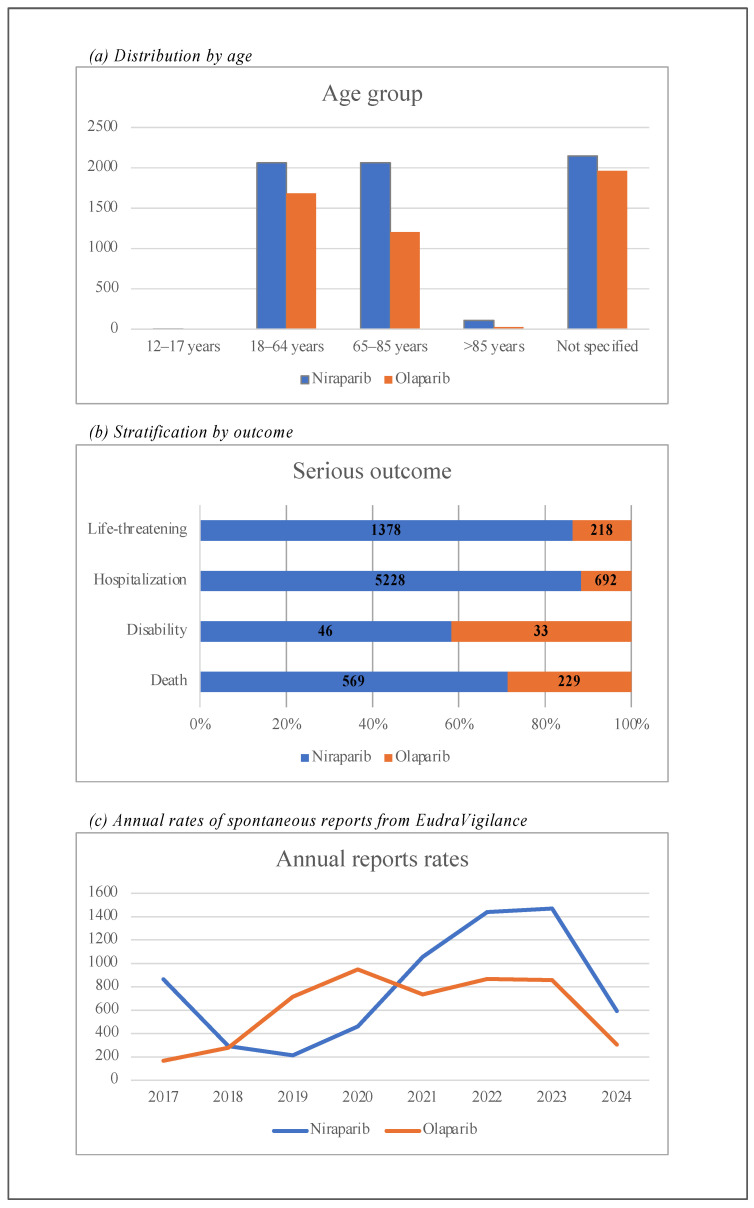
Distribution of individual case safety reports (ICSRs) by age (**a**), severity (**b**), and years (**c**).

Figure 2 showcases graphical summaries of the ICSRs’ distribution across age, outcome, and years related to NIRA and OLA from the EV database.

In terms of severity, 58.7% of NIRA-related ADRs were classified as non-serious, compared to 30.8% for OLA. According to the definition of serious adverse events (SAEs), the percentages of ADRs reporting outcomes such as death, disability, hospitalization, and life-threatening conditions for NIRA and OLA are as follows: death (8.9% NIRA; 4.7% OLA), disability (0.72% NIRA; 0.67% OLA), hospitalization (81.88% NIRA; 14.2% OLA), and life-threatening conditions (21.58% NIRA; 30.8% OLA). In accordance with EMA recommendations, data were grouped into the following age categories: 12–17 years (*n* = 1 NIRA), 18–64 years (*n* = 2063 NIRA; *n* = 1682 OLA), 65–85 years (*n* = 2061 NIRA; *n* = 1206 OLA), over 85 years (*n* = 108 NIRA; *n* = 27 OLA), and not specified (*n* = 2149 NIRA; *n* = 2745 OLA).

**Figure 3:**
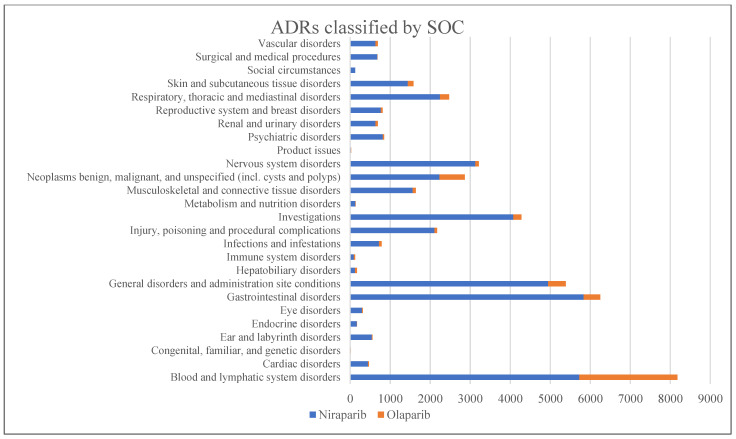
Frequency of adverse drug reactions (ADRs) classified by System Organ Class (SOC).

The frequency of ADRs classified by System Organ Class (SOC) for NIRA and OLA is shown in Figure 3.

The disproportionality analysis was performed through the calculation of the Relative Risk (RR) with a 95% confidence interval (CI).

**Figure 4:**
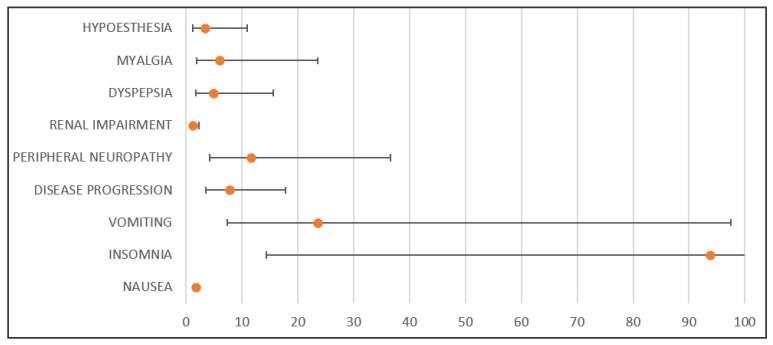
Disproportion analysis of the most significant signals related to niraparib compared to olaparib. The orange circles represent the Relative Risk (RR), and the horizontal black lines represent the 95% CI for RR.

NIRA-related ADRs that were more likely to be reported than ADRs in the comparison PARPi included: thrombocytopenia (*n*: 2447; RR: 1.72; 95% CI: 1.48–1.99; *p* < 0.001), nausea (*n*: 1497; RR: 1.69; 95% CI: 1.40–2.05; *p* < 0.01 ), anemia (*n*: 1335; RR: 8.96; 95% CI: 8.38–9.58; *p* < 0.001), fatigue (*n*: 1229, RR: 2.31, CI: 1.80–2.95; *p* < 0.001), constipation (*n*: 874; RR: 12.75; 95% CI: 96.44–26.21; *p* < 0.001), insomnia (*n*: 714; RR: 93.74; 95% CI: 14.42–179.80; *p* < 0.001), headache (*n*: 597; RR: 7.13; 95% CI: 3.83–13.62; *p* < 0.001), blood pressure increase (*n*: 574; RR: 18.84; 95% CI: 6.82–59.02; *p* < 0.001), vomiting (*n*: 538; RR: 23.54; 95% CI: 7.37–97.46; *p* < 0.001), decreased appetite (*n*: 512; RR: 1.72, 95% CI: 1.23–2.42), product dose omission issues (*n*: 508; RR: 66.69; 95% CI: 10.25–1279.93), leukopenia (*n*: 465; RR: 1.11; 95% CI: 085–1.48; *p* = 0.49), carbohydrate antigenic 125 increase (*n*: 458; RR: 1.67; 95% CI: 1.18–2.38; *p* = 0.002), neutropenia (*n*: 455; RR: 0.29; 95% CI: 0.24–0.34; *p* < 0.001), dyspnea (*n*: 441; RR: INF; 95% CI: 12.07-INF; *p* < 0.001), dizziness (*n*: 419; RR: 4.23; 95% CI: 2.39–7.68; *p* < 0.001), ovarian cancer (*n*: 415; RR: 27.24; 95% CI: 6.73–157.82), disease progression (*n*: 412; RR: 7.73; 95% CI: 3.56–17.75; *p* < 0.001), off-label use (*n:* 409; RR: 3.16; 95% CI: 1.91–5.30; *p* < 0.001), hypertension (*n:* 398; RR: 1.34; 95% CI: 0.96–1.89; *p* = 0.08), erythropenia (*n*: 371; RR: 6.09; 95% CI: 2.94–13.20; *p* < 0.001), peripheral neuropathy (*n*: 355; RR: 11.65; 95% CI: 4.21–36.59; *p* < 0.001), recurrent ovarian cancer (*n*: 338; RR: 0.91; 95% CI: 0.67–1.24; *p* = 0.906), heart rate increase (*n*: 316; RR: INF; 95% CI: 8.65-INF; *p* < 0.001), malaise (*n*: 308; RR: 0.96; 95% CI: 0.69–1.35; *p* = 0,96), malignant neoplasm (*n*: 306; RR: 7.99; 95% CI: 6.83–9.35; *p* < 7.99), renal impairment (*n*: 297; RR: 1.15; 95% CI: 0.99–2.29; *p* = 0.045), pain (*n*: 279; RR: 36.63; 95% CI: 5.62–703.98; *p* < 0.001), arthralgia (*n:* 279; RR: 0.80; 95% CI: 0.59–1.10; *p =* 0.80), diarrhea (*n*: 232; RR: 0.74; 95% CI: 0.53–1.05; *p* = 0.087), recurrent cancer (*n*: 229; RR: 30.07; 95% CI: 4.60–578.22; *p* < 0.001), condition aggravation (*n:* 224; RR: 14.70; 95% CI: 3.62–85.43; *p* < 0.001), myelosuppression (*n*: 213; RR: 0.30; 95% CI: 0.24–0.40; *p* < 0.001), drug ineffectiveness (*n*: 207; RR: 0.85; 95% CI: 0.58–1.25; *p* = 0.363), weight decrease (*n*: 207; RR: 27.32; 95% CI: 3.83–194.87; *p* < 0.001), anxiety (*n*: 206; RR: 4.51; 95% CI: 1.94–11.23; *p* < 0.001), dry mouth (*n*: 201; RR: INF; 95% CI: 5.49-INF; *p* < 0.001), back pain (*n*: 201, RR: 13.19; 95% CI: 3.25–76.72; *p* < 0.001), palpitations (*n*: 196; RR: INF; 95% CI: 5.35-INF; *p* < 0.001), rash (*n:* 195; RR: 1.83; 95% CI: 1.04- 3.28; *p =* 0.023), decreased activity (*n:* 191; RR: INF; 95% CI: 5.22-INF; *p* < 0.001), dry mouth (*n*: 191; RR: INF; 95% CI: 5.22-INF; *p* < 0.001), feeling abnormal (*n*: 187; RR: INF; 95% CI: 5.11-INF; *p* < 0.001), ADR (*n*: 181; RR: 2.64; 95% CI: 1.32–5.504; *p* = 0.002), pain in extremities (*n*: 180; RR: 7.88; 95% CI: 2.45–30.81; *p* > 0.001), increased blood creatinine (*n*: 174; RR: 0.47; 95% CI: 0.34–0.65; *p* < 0.001), hospitalization (*n:* 168; RR: INF; 95% CI: 4.58-INF; *p* < 0.001), an abnormal laboratory test (*n:* 160; RR: 5.25; 95% CI: 1.88–16.61; *p* < 0.001), contusion (*n*: 159; RR: 6.96; 95% CI: 2.16–27.25; *p* < 0.001), abnormal full blood count (*n*: 157; RR: 10.31; 95% CI: 2.53–60.04; *p* < 0.001), pruritus (*n*: 155; RR: 2.54; 95% CI: 1.21–5.58; *p* = 0.005), dehydration (*n*: 152; RR: 3.99; 95% CI: 1.58–11.00; *p* < 0.001), dyspepsia (*n*: 150; RR: 4.92; 95% CI: 1.76–15.58; *p* < 0.001), death (*n*: 144; RR: 0.40; 95% CI: 0.29–0.57; *p* < 0.001), illness (*n*: 143; RR: 6.26; 95% CI: 1.94–24.54; *p* < 0.001), stomatitis (*n*: 142; RR: 4.66; 95% CI: 1.67–14.77; *p* < 0.001), urinary tract infection (*n*: 141; RR: INF; 95% CI: 3.84-INF; *p* < 0.001), myalgia (*n*: 137; RR: 6.00; 95% CI: 1.85–23.52; *p* < 0.001), product dose omission error (*n*: 133; RR: INF; 95% CI: 3.62-INF; *p* < 0.001), inappropriate antidiuretic hormone secretion (*n*: 111; RR: INF; 95% CI: 3.02-INF; *p* < 0.001), peripheral swelling (*n*: 111; RR: INF; 95% CI: 3.02-INF; *p* < 0.001), inappropriate schedule for product administration (*n:* 110; RR: 14.44; CI 95%: 2.20–278.94; *p* < 0.001), gait disturbance (*n*: 106: RR: 6.96; CI 95%: 1.70–40.72; *p* < 0.001), hypoesthesia (*n*: 104; RR: 3.41; CI 95%: 1.21–10.87; *p* = 0.006), and muscle spasm (*n*: 103; RR: 4.512; 95% CI: 1.39–17.78; *p* = 0.002). The disproportion analysis of the most significant signals related to NIRA compared to OLA is shown in Figure 4.

**Figure 5:**
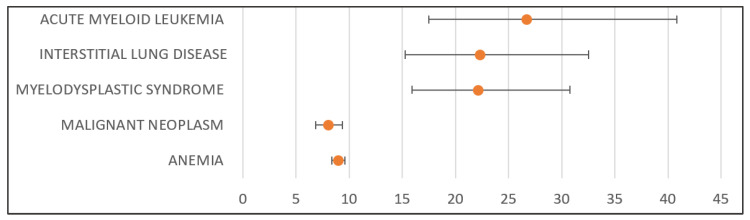
Disproportion analysis of the most significant signals related to olaparib compared to niraparib. The orange circles represent the Relative Risk (RR), and the horizontal black lines represent the 95% CI for RR.

Compared to NIRA, the probability of reporting OLA-related ADRs was higher for: anemia (*n*: 1571; RR: 8.96; CI 95%: 8.38–9.58; *p* < 0.001), malignant neoplasm (*n*: 321; RR: 7.99; CI 95%: 6.83–9.35; *p* < 0.001), MDS (*n*: 148; RR: 22.10; CI 95%: 15.93–30.75; *p* < 0.001), interstitial lung disease (*n*: 114; RR: 22.27; CI 95%: 15.29–32.54; *p* < 0.001), and AML (*n*: 105; RR: 26.66; CI 95%: 17.50–40.83; *p* < 0.001). Figure 5 presents the disproportionality analysis of the main significant signals for OLA in comparison to NIRA.

## 3. Discussion

Our real-world data analysis, based on data from the EV database from 2017 to 2024, examined case reports of suspected ADRs associated with OLA and NIRA, two PARPi used primarily to treat ovarian cancer. Niraparib was associated with a higher percentage of serious ADRs (49.84%) and hospitalizations (81.88%) than OLA (53.00% and 14.20%, respectively), suggesting that NIRA-related AEs tend to be more serious. In addition, life-threatening ADRs were reported more frequently in patients treated with NIRA (21.58%) than with OLA (4.47%). However, NIRA had a higher proportion of non-serious ADRs (58.72%) than OLA (30.80%), suggesting that a significant proportion of ADRs with both drugs were mild in nature. The mortality rate was also higher for NIRA (8.90%) compared to OLA (4.70%), with more deaths reported in patients receiving NIRA. This trend suggests that, although both drugs are associated with serious AEs, NIRA may carry a higher clinical risk, particularly in terms of life-threatening outcomes and mortality. Furthermore, the age distribution for both drugs shows that the majority of reports were from adults aged 18–64 years, with the highest reporting years being 2023 for both drugs. The descriptive analysis of SOCs shows that the highest frequency of ADRs for both drugs was in the category of disorders of the blood and lymphatic system. Specifically, 5725 ADRs related to NIRA and 2457 related to OLA were reported in this category, indicating a significant incidence of hematological AEs. Other notable SOCs with high frequencies for NIRA included gastrointestinal disorders (5835 ADRs), general disorders and administration site conditions (4946 ADRs), and nervous system disorders (3127 ADRs). In comparison, OLA had lower frequencies in these categories, with 415 reports for gastrointestinal disorders, 441 for general disorders, and 85 for nervous system disorders. However, OLA had a higher relative incidence of benign, malignant, and unspecified neoplasms (634 reports for OLA vs. 2229 for NIRA) and endocrine disorders (2 reports for OLA vs. 163 for NIRA). Cardiac disorders were significantly more common with NIRA (442 reports) than with OLA (27 reports).

In addition, NIRA was associated with higher frequencies in categories such as infections and infestations (717 reports), musculoskeletal and connective tissue disorders (1560 reports), psychiatric disorders (815 reports), and respiratory, chest, and mediastinal disorders (2244 reports). OLA had very low numbers in categories such as cardiac disorders (27 reports) and congenital, hereditary, and genetic disorders (7 reports). Overall, NIRA shows a broader and more severe ADR profile across several organ systems, with gastrointestinal, blood, and nervous system disorders being the most prominent. Olaparib shows a lower incidence of AEs in these categories, with the exception of neoplasms and some endocrine disorders.

The most frequently reported ADRs for both drugs were nausea, fatigue, anemia, vomiting, neutropenia, and thrombocytopenia, although each drug had a lower incidence.

For NIRA, the most common reactions were thrombocytopenia (2447 cases), nausea (1497 cases), fatigue (1229 cases), and constipation (874 cases), with high RR values particularly for constipation, insomnia, and increased blood pressure. The results suggest that thrombocytopenia and anemia are among the most common and severe reactions, consistent with data from clinical trials. Some adverse reactions, such as peripheral neuropathy and dyspepsia, were reported more frequently than documented in clinical trials, which may indicate a higher incidence of adverse reactions in routine clinical practice compared to RCTs that mainly include selected patient populations.

For OLA, the most common ADRs were anemia, malignant neoplasm, MDS, and AML. The incidence of MDS and AML was significantly higher than observed in clinical trials, suggesting a stronger association between OLA and these events. The data also show a high risk of interstitial lung disease, a rare but serious event that has been reported.

Currently, there are no robust data in the literature comparing results from registration trials with those from real-world studies. By analyzing the long-term outcomes of PARPi for OC, Turinetti and colleagues underlined a higher incidence of AML and MDS, from 0.2% to 6.6% for NIRA and from 1.5% to 8.2% for OLA, thereby emphasizing the importance of real-world data in further defining both the safety and the efficacy of PARPi [25].

## 4. Material and Methods

This retrospective pharmacovigilance study was conducted using the EudraVigilance (EV) database (web site https://www.adrreports.eu/it/index.html, accessed on 30 March 2025). Olaparib and niraparib related ICSRs from 1 January 2017 to 30 April 2024 were retrieved. ADRs were classified using the MedDRA^®^26.0 dictionary. The selection process was conducted at the case level using the identification number. Data were evaluated using descriptive and disproportionality analyses. Descriptive analyses were performed, stratifying the data by age, seriousness of ADRs, and Preferred Terms (PTs). Relative Risks (RRs), along with their 95% confidence intervals (CIs), were used as a measure of disproportionality. Additionally, a brief review of the literature on the safety of OLA and NIRA was conducted, including data from six RCTs and two phase II trials.

## 5. Conclusions

Spontaneous reporting system analyses are among the most widely used and effective pharmacovigilance methods for generating potential signals that warrant further investigation and validation [26]. The EV database, with its comprehensive collection of reports, provides valuable insight into ADRs associated with the use of PARPi approved for the treatment of ovarian cancer, including rare and serious events. Given the increasing use of PARPi, there is an urgent need to investigate the safety of these drugs, particularly regarding hematological ADRs. The main strength of this study lies in its contribution to the growing knowledge on the safety profiles of OLA and NIRA, using a European database and applying disproportionality analysis. However, as data in ICSRs are derived from spontaneous reports, they may be incomplete or of variable quality, often lacking essential information such as clinical characteristics, health status, concomitant medications, comorbidities, outcomes, and follow-up details [27]. One must also consider the tendency to believe that target therapies cause fewer adverse reactions, which may lead to under-reporting, particularly by healthcare professionals [28].

The objectives of this study were (i) to characterize the adverse reactions associated with OLA and NIRA as reported and recorded in the EV database, (ii) to compare the frequency of the ADRs occurring during treatment with one of the two PARPi, and (iii) to compare post-marketing safety data with data from clinical trials.

This analysis suggests that both drugs exhibit a safety profile consistent with clinical trial evidence, yet with a higher frequency of AEs observed in real-world clinical practice. Specifically, an increased incidence of peripheral neuropathy has been reported in patients treated with NIRA. Tian and colleagues had previously hypothesized that such occurrences might be more frequent in patients receiving this PARPi [29]. Both OLA and NIRA have similar safety profiles, with common AEs including nausea, vomiting, anemia, thrombocytopenia, and fatigue. However, OLA is associated with a higher risk of MDS, AML, and interstitial lung disease, while NIRA shows a higher incidence of gastrointestinal events and thrombocytopenia. These differences in safety profile suggest that the choice between the two drugs should be based on a careful assessment of individual patient risk, particularly for those with a predisposition to hematological or pulmonary complications. Continuous monitoring and individualized management of adverse effects are essential to optimize the benefits of treatment. However, additional real-world studies are needed to further investigate these ADRs and their impact on patients’ clinical outcomes, especially in terms of quality of life.

## Figures and Tables

**Table 1:** Niraparib safety profile from registration studies.

Study	AE	Any Grade	Grade ≥3
PRIMA [9]	Thrombocytopenia	67.1%	39.7% 31.6 %1.2% 21.3% 0.4%
	Anemia	65.1%	
	Nausea	58.3%	
	Neutropenia	43.2%	
	Constipation	41.7%	
NORA [18]	Fatigue	36.6%	2.3%
	MDS/AML	1.2%	
	Platelet count decreased	54.8%	11.3%
	Anemia	53.1%	14.7%
	Nausea	53.1%	0%
	Vomiting	32.2%	2.3%
	Constipation	29.9%	0.6%
QUADRA [21]	Nausea	58%	4%
	Fatigue	41%	5%
	Anemia	44%	24%
	Vomiting	32%	4%
	Thrombocytopenia	33%	21%
	Neutropenia	7%	11%
	Constipation	17%	1%
	Platelet count decreased	21%	13%
	Small intestinal obstruction	7%	

Note: AE, adverse event; MSD, myelodysplastic syndrome; AML, acute myeloid leukemia.

**Table 2:** Olaparib safety profile from registration studies.

Study	AE	Any Grade	Grade ≥3
SOLO 1 [7,8]	Nausea	77.7%	0.8%
	Fatigue	64.2%	3.8%
	Anemia	40%	21.9%
	Vomiting	40%	0.4%
	Neutropenia	23.1%	8.5%
	Diarrhea	34.6%	3.1%
	Other tumors	5.4%	
SOLO 2 [5]	Nausea	75.9%	3%
	Fatigue	65.5%	4%
	Anemia	43.6%	19%
	Vomiting	37.4%	3%
	Neutropenia	19%	5%
	Diarrhea	32.8%	1%
	Other tumors	4.5%	
SOLO 3 [23]	Nausea	64.6%	1.1%
	Fatigue	52.5%	4.5%
	Vomiting	38.2%	1.1%
	Diarrhea	28.1%	
	Abdominal pain	21.3%	1.1%
	Headache	15.7%	
	Constipation	12.4%	
	Anemia	51.1%	21.3%
	Neutropenia	23%	9.6%
	Thrombocytopenia	11.8%	3.9%
	MDS/AML	2%	
	Other tumors	1.7%	
LIGHT [24]	Nausea	66.4%	1.8%
	Fatigue	62%	4.1%
	Vomiting	32.8%	1.1%
	Anemia	28.8%	15.1%

Note: AE, adverse event; MSD, myelodysplastic syndrome; AML, acute myeloid leukemia.

## Data Availability

The datasets presented in this study can be found in online repositories. The names of the repository can be found below: https://www.adrreports.eu/en/index.html, accessed on 30 March 2025.
